# Development and validation of the short version of the Psychological General Well-Being Index (PGWB-S)

**DOI:** 10.1186/1477-7525-4-88

**Published:** 2006-11-14

**Authors:** Enzo Grossi, Nicola Groth, Paola Mosconi, Renata Cerutti, Fabio Pace, Angelo Compare, Giovanni Apolone

**Affiliations:** 1 Medical Department Bracco SpA, Milan, Italy; 2 Novartis Vaccines and Diagnostics SRL, Siena, Italy; 3 Institute of Pharmacological Research, Mario Negri, Milan, Italy; 4 Data Management and Statistics Unit, Bracco SpA, Milan, Italy; 5 Gastrointestinal Unit, L. Sacco Hospital, Milan, Italy; 6 Psychology Department, Catholic University, Milan, Italy; 7 Italian Institute of Auxology, Milan, Italy

## Abstract

**Background:**

The PGWBI is a 22-item health-related Quality of Life (HRQoL) questionnaire developed in US which produces a self-perceived evaluation of psychological well-being expressed by a summary score. The PGWBI has been validated and used in many countries on large samples of the general population and on specific patient groups. Recently a study was carried out in Italy to reduce the number of items of the original questionnaire, yielding the creation of a shorter validated version of the questionnaire (PGWB-S). The purpose of the present paper is to describe the methods adopted and to report and discuss the relevance of results.

**Methods:**

Data for this study were collected from 4 different population samples: two general population samples a student and a patient sample. On the basis of the results of the first (development) sample population, six relevant items were identified statistically from the original questionnaire and grouped to assemble a new summary scale. Following the newly created 6-item questionnaire was administered in three independent population samples. Descriptive statistics, correlation coefficients, univariate and multivariate regression analyses were used to compare the performance of the long and short questionnaire, within and between population samples and across relevant subgroups. A further independent sample extracted by an ongoing cancer clinical trial served as final validation step.

**Results:**

Overall, the questionnaires were administered to 1443 subjects. Six items were selected by a step-wise approach to explain 90% of the variance of the summary measure of the original questionnaire.

Response rates reached 100%, while missing items were not observed. University students (n = 400) showed the highest mean value of the summary measure (75.3); while the patient sample (n = 28) had the lowest score (71.5). The correlation coefficients between the summary measures and the single items according to the different studies were satisfactory, reaching the highest estimates in the student sample. The internal consistency showed high values of the Cronbach's alpha coefficient (range 0.80 – 0.92) for all three study samples, coming close to the value of the coefficient established for the original questionnaire (0.94). A cross-validation in an independent sample of 755 cancer patients confirmed the item selection procedure and amount of variance explained by the new shorter questionnaire (ranging from 90. 2 to 95.1 %, across age and sex strata).

**Conclusion:**

The newly identified PGWB-S showed good acceptability and validity for the use in various settings in Italy. The translation of the PGWB-S into different languages, and its use in other linguistic settings will add evidence about its cross-cultural validity.

## Background

The PGWBI questionnaire is a validated Health Related Quality of Life (HRQoL) measure, widely used in clinical trials and epidemiological research to provide a general evaluation of self-perceived psychological health and well-being [1-10]. In the late sixties Harold Dupuy, psychologist at the National Center for Health Statistics, developed his Psychological General Well Being Schedule, a questionnaire of 68 items to measure the degree of 'happiness' of the American population or the potential psychological distress. The questionnaire was considered one of the first generic measures of health-related quality of life with specific interest to mental health.

Some years after Dupuy together with John E. Ware revised the questionnaire and a final version of 22 selected items was validated under the name of PGWB Index (PGWBI). Extensive reference data of this version generated in the US general and patient population were published and fully described in 1984 in "Assessment of Quality of Life in Clinical Trials of Cardiovascular Therapies" [11].

About ten years after the index was also introduced in Europe. The PGWBI was adapted in many languages and cross-culturally validated for the use in several countries under the coordination of the MAPI Research Institute. As a result different language versions of the PGWBI are available for use on the MAPI website [12].

In Italy various research activities concerning the field of Outcome Research were started in the framework of the MiOS project, a multidisciplinary initiative to study in depth different kinds of subjective outcome measures for the health assessment. In 2000 as part of the MiOS project, the PGWBI was validated in a representative sample of 1129 Italian citizens above 15 years of age. The results of this study were published of the Italian user manual and were recognized as reference data for the self-perceived health in the Italian general population [13-15]. During the same period the MiOS group validated also the Italian version of the Short Form-12 (SF-12) derived from the original longer version of the SF-36 [16,17]. The successful validation of the SF-12 set the ground for the development of an abbreviated and more user-friendly version of the original PGWBI. The reason for the reduction of items was to achieve a higher acceptability of the questionnaire in the population, aiming for shorter times of administration, better response rates and lower rates of missing data.

The main objective of this study was to reduce the number of items of the original 22-item PGWBI while keeping adequate validity and reliability of the questionnaire. The step-wise approach to identify the best and most relevant set of items and the results of the application of the new PGWB-Short (PGWB-S) in various settings, including samples of general and specific patient populations, are described in this paper.

## Methods

### Development and validation strategy of the PGWB-S

The original PGWBI consists of 22 self-administered items, rated on a 6-point scale, which assess psychological and general well-being of respondents in six HRQoL domains: anxiety, depressed mood, positive well-being, self-control, general health and vitality [see Additional file 1]. Each domain is defined by a minimum of 3 or a maximum of 5 items. The scores for all domains can be summarized to provide a summary score, which reaches a maximum of 110 points, representing the best achievable "well being".

Item reduction for the development of the short version of the PGWBI was started from the reference data set achieved during the year 2000 when the original (long) questionnaire was administered for the first time in Italy to a representative sample of the general population (development sample). The survey was carried out by DOXA, the Italian branch of the Gallup International association. Methods and results are available elsewhere [13-15].

Based on these data, the twenty-two items of the questionnaire were analyzed in a linear multiple regression model with the objective to find the best combination of items to be most relevant for the determination of the summary score. For comparability purpose with the longer version, a score transformation was applied to convert the lowest and highest possible scores to 0 (worst possible level of well-being) and 110 (maximum level of well being), respectively.

The new shorter questionnaire was then administered in three different settings in Italy for the purpose of its further validation. All studies took place during the year 2004 and their characteristics are summarized in Table 1.

**Table 1:** Characteristics of studied samples

**Study**	**Development STUDY**	**Study 1**	**Study 2**	**Study 3**
Organization, Location	DOXA, MILAN	DOXA, MILAN	CATHOLIC UNIVERSITY, MILAN	UNIVERSITY HOSPITAL SACCO,MILAN
Year	2000	2004	2004	2004
# cases	1129	1015	400	28
Questionnaire administered	PGWBI	PGWB-S	PGWBI, PGWB-S	PGWBI, PGWB-S
Sampling method	random	random	random	random
Population	General population	General population	University students, in second year of Psychology and others	In-patients with diagnosis of chronic inflammatory bowel disease
Mode of administration	Person-to-person*	Person-to-person*	Self-administered**	Self-administered**
Male %	48.1	49.5	11.4	39.3
Age, mean yrs	47.4	51.3	21.5	50.1

* Self-administration in a structured interview

** Self-administration of both questionnaires one hour apart (cross-over design)

### Development and validation samples

#### Study 1 (general population)

In 2004 a cross-sectional survey was carried out again by DOXA to norm the new short version of the questionnaire in a representative sample (n = 1015) of community-dwelling Italians. The approach used was similar to the one implemented in the previous DOXA survey [13-15]. A multi-step random sampling method was adopted to draw a large representative sample from the Italian population. The universe, to which the National survey referred, were 49.2 million Italians of all regions aged 15 years or more, stratified according to region and size of the place of residence. The sampling units were chosen in the following way: in the first stage, the choice regarded the municipalities where the interviews were to be conducted, in the second stage in each municipality an adequate number of electoral wards were extracted at random so that various types of urban areas were represented (e.g., central, suburban, outskirts and isolated houses), finally, names and addresses of the persons to be contacted were extracted at random from the electoral lists of the areas selected in the second stage. Mean scores for all items and the global summary measures were calculated according to the established algorithm and weighted by gender, age and size of the municipality in the percentages as established in the universe which the study referred to.

#### Study 2 (student population)

The purpose of this class room experiment was to determine the self-perceived psychological and general well-being of a random sample of students in the second year of Psychology (n = 246) at the Catholic University of Milan. Additional 154 students in the second year of Faculty of Motor Sciences were included into the sample. For the purpose of comparison the original PGWBI and the new PGWB-S were self-administered by all students one hour apart. The order of questionnaires (long and short form) was randomly allocated and summary scores of both questionnaires were then compared.

#### Study 3 (patient population)

The study was performed in the hospital ward of Gastroenterology and Rheumatology in the Sacco Hospital in Milan. Twenty eight patients suffering from chronic inflammatory bowel disease were enrolled into the study. Both questionnaires were self-administered in the context of a planned medical visit and items and summary measures were calculated.

### Analysis

Because the goal was to identify the best set of items that might reproduce the summary score of the longer version, we first selected the items in the development sample (DOXA 2000) using a multiple step-wise regression procedure: the goal was to select the minimum number of items that might explain at least 90% of the variance of the original longer (22 item) questionnaire. According to the previous experience in the context of the development and cross-cultural validation of the SF-12 [17-19], items were identified by a step-wise selection starting with the item that would give alone the highest degree of variance of the original SF-36, adding items until their combination would explain at least 90% of the variance. In the model the items were matched to find out which of their combination would best reproduce the mean value of the summary score. The most predictive items were selected to be part of the new structure of the questionnaire PGWB-S and then aggregated in a new summary score. Following, the performance of the new shorter questionnaire was assessed in an additional DOXA sample and in two other independent settings.

As emphasised in the literature [18,20-22], great care was taken to ensure and document the basic characteristics of the questionnaire in terms of acceptability, internal consistency (Cronbach's alpha coefficient), known-group validity and stability of results across samples and sub-groups. Descriptive statistics, correlation coefficients, univariate and multivariate regression analyses were used to evaluate the performance of the long and short questionnaire, in each sample and across relevant subgroups.

## Results

**Figure 1:**
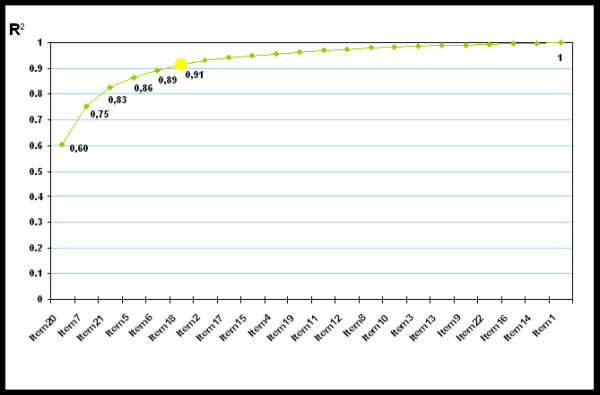
PGWBI short: items selection.

During the step-wise selection process six items were identified to predict 90% variance of the summary score when the original long questionnaire was applied to a random sample of the Italian population. Item 20 alone reached 60%, whereas Items 7, 21, 5, 6, 18 and 2 added an additional 15%, 8%, 3%, 3% and 2%, respectively (Table 2 and Figure 1). These items were confirmed to become part of the new 6-item structure of the questionnaire.

**Table 2:** Selection of items in the PGWB-S

**ITEMS PGWB-S**	**Dimension**	**Position in the Questionnaire**	**Content**
Item 05	Anxiety	5	Have you been bothered by nervousness or your "nerves" during the past month?
Item 06	Vitality	6	How much energy, pep, or vitality did you have or feel during the past month?
Item 07	Depressed mood	7	I felt downhearted and blue during the past month.
Item 18	Self-control	18	I was emotionally stable and sure of myself during the past month.
Item 20	Positive well-being	20	I felt cheerful, lighthearted during the past month.
Item 21	Vitality	21	I felt tired, worn out, used up, or exhausted during the past month.

In order to evaluate the performance of the new instrument, one thousand four hundred and forty three subjects were evaluated in the different settings. Socio-demographic characteristics varied accordingly to the case-mix evaluated: the mean age ranged from 21.5 years in the study involving University students to 51.3 years for the sample representing the general Italian population. The gender distribution ranged from 11 % to 50 % of males among the studies (Table 1).

The step-wise selection process previously applied to Study 1 confirmed the relevance of the six items identified in the development sample of the previous DOXA Study. Six items predicted 88% variance of the summary score when the long questionnaire was applied to Study 2 sample. Item 20 alone reached 55%, whereas Items 21, 7, 6, 5, 18 and added an additional 11%, 9%, 6%, 4% and 3%, respectively.

As to the acceptability indicators, response rates of satisfactory 100% were reached in all studies (Table 1). No missing and out-of-range data at item level were registered for any of the samples.

Descriptive statistics of raw item scores are presented in Table 3 according to the different studies. As expected, the sample of the University students reported the highest mean value of the summary measure (75.3; range 44–106), while the lowest was reported by the sample of hospital patients with chronic inflammatory bowel disease (71.5; range 63.7–79.4). In the sample of the University students where both versions of the questionnaires were self-administered in a cross- over design, the mean value of the summary score of the long form (74.8; range 41–105) confirmed the results of the short form. The subjective mental health perception was measured without specific relation to the perceived physical health, therefore overall values did not show differences between groups and were relatively stable across studies.

**Table 3:** Mean values of PGWB-S Items and Summary scores according to studies

ITEMS PGWB-S	***Study 1***	***Study 2***	***Study 3***
Item 05	12.81	16.09	16.39
Item 06	12.70	11.65	8.61
Item 07	13.14	12.29	11.96
Item 18	10.94	11.04	12.14
Item 20	10.43	11.74	10.21
Item 21	12.45	12.48	12.25

Summary score	72.46 (18.26)	75.28 (14.32)	71.50 (20.26)

The patterns of correlation between the summary scores and the single items according to the different studies are presented in Table 4. Subjects in the general population had the lowest correlation between the single item and the summary score, whereas the highest correlation with the summary score was observed for the items of the University students. The lowest correlation estimates in all studies was observed consistently for 'item 18' regarding the question on self-control (range 0.52 – 0.72), whereas the highest correlation (0.89) was observed in study 2 for 'item 05' regarding anxiety.

**Table 4:** Correlations of Item and Summary scores according to studies^1^

ITEMS PGWB-S	***Study 1***	***Study 2***	***Study 3***
Item 05	0.04	0.06	0.04
Item 06	0.04	0.04	0.04
Item 07	0.04	0.06	0.05
Item 18	0.04	0.05	0.04
Item 20	0.05	0.06	0.05
Item 21	0.04	0.06	0.04

1Figures in the tables represent standardized regression coefficients estimated using a multivariable regression model having the summary measure as dependent variables and items as independent

The internal consistency measuring the extent to which the items are interrelated were expressed by the coefficients Cronbach's Alpha calculated for each study. Table 5 shows the coefficients Cronbach's Alpha obtained for the PGWBI and the PGWB-S in the individual study settings. The smallest value was 0.80 and the highest 0.92, indicating that the summary score showed good internal reliability. The coefficients Cronbach's Alpha were all above 0.80 showing acceptable reliability, also when compared to the one (0.94 in DOXA Study and 0.96 in Study 2) of the original instrument in full length (22 items). Finally, Table 6 reports the sex-adjusted summary scores for the age groups in study 1 when compared to the study in which the original PGWBI was administered. Mean values of the summary scores decreased with age ranging from 85.4 in the young to 71.7 in the elderly and 81.8 to 63.9 in the individual studies. The impact of ageing on the self-perceived mental health can be observed. In the past, a clear age trend has been documented for physical health measures, while the mental health measures have shown to be less sensitive to the age effect [13,16,17]. The raw summary scores given for study 2 and 3 although referring to restricted age groups and small sample sizes fitted into the overall age trend observed in the two large field trials in the Italian population.

**Table 5:** Cronbach's Alpha Coefficients of summary scores according to studies

	**DOXA**	**Study 1**	**Study 2**		**Study 3**
	**PBWBI**	**PGWB-S**	**PGWBI**	**PGWB-S**	**PGWB-S**
**Cronbach alpha**	0,07	0,06	0,07	0,06	0,06

**Table 6:** Mean values of summary scores according to age and study^1^

**Age (years)**	**DOXA**	**Study 1**	**Study 2**		**Study 3**
	**PBWBI**	**PGWB-S**	**PGWBI**	**PGWB-S**	**PGWB-S**

15–17	83.5 (16.95)	81.8 (19.07)	NA	NA	NA
18–20	85.4 (17.61)	81.5 (16.46)	NA	NA	NA
21–24	81.9 (17.30)	78.4 (16.07)	74.8 (16.13)	75.3 (14.31)	NA
25–29	80.3 (16.95)	77.0 (18.16)	NA	NA	NA
30–34	80.2 (16.40)	70.9 (16.69)	NA	NA	NA
35–39	76.4 (15.0)	71.0 (19.80)	NA	NA	NA
40–44	78.7 (16.0)	70.0 (15.10)	NA	NA	NA
45–49	81.1 (12.66)	70.5 (17.80)	NA	NA	NA
50–54	74.1 (18.97)	69.7 (18.20)	NA	NA	71.5 (20.26)
55–64	75.6 (17.93)	70.6 (18.41)	NA	NA	NA
65–74	71.7 (20.74)	71.8 (18.85)	NA	NA	NA
≥ 75	73.0 (24.68)	63.9 (21.05)	NA	NA	NA

1 Figures in the table represent means and standard deviations of gender-adjusted scores

NA Not available

## Discussion

The extensive international experience with the original PGWBI and the many data generated in recent years [23-30] were the basis on which it has been possible to perform a meaningful item reduction resulting in the development of a new shorter instrument in Italy. The aim of this study was to identify the lowest number of items, which would be sufficient to maintain the validity of the original questionnaire. We identified 6 items that reproduced at least 90% of the variance of the PGWB summary Index through multiple step-wise regression analysis.

Compared to the PGWBI, where the global summary score is generated by summing up 22 items pertaining the six subscales, the new PGWB-S is constructed on the basis of only six items representing five of the six original subscales. When tested in various samples of the Italian population the acceptability (response rate, missing data) and validity of the PGWB-S demonstrated a satisfactory performance of the PGWB-S across strata. The good compliance expressed by absence of missing data was probably favoured by the structured person-to-person interviews in study 1 or the self-administration in controlled settings in study 2 and 3. Nevertheless it is worth mentioning that the relatively short time necessary to answer the six questions of the questionnaire might have contributed positively to this result.

With respect to validity, the global summary scores varied across the different groups, reflecting the expected degree of variation related to the baseline characteristics of the participants.

The relatively high values of the Cronbach's alpha coefficients observed in the samples indicate a good reliability of the questionnaire when compared to the Gold Standard of the original PGWBI. In spite of the slightly lower precision of the 6-item questionnaire in comparison with the original, the PGWB-S came out to be a robust instrument, suitable as a generic measure of HRQoL and a good tool for population surveys, where it can be easily administered.

Our study has a few limitations that should be considered.

The first pertains to the method adopted to select the relevant items. Alternatives methodologies are indeed often used alone or in combination for this purpose, such as item-total correlations using Cronbach's alpha coefficients, and principal components factor analysis. Our choice was essentially based on the experience on the development of the SF-12 that has the advantage to be straightforward, easy to be replicated and comprehensive to be understood by lay people. On the other hands, we cannot exclude that other methods could yield different outputs. One might also argue that present results, in terms of item selection and performance of the new shorter index might be result of the specific characteristics of the development and validation samples. Waiting for further independent validation of our exercise, in order to add information about the performance of the new questionnaire, we further tested the robustness of our findings by performing a cross-validation in an independent sample of 755 cases, extracted from an on-going clinical trial where the original (long) PGWBI was used together with other patient-reported measures [31]. In this data set, we first replicated the step-wise item procedure to cross-validate the selection of the PGWB-S and then estimated how well the PGWB-S developed in the original DOXA sample would explain the variance of the longer 22 item questionnaire. As to the item selection, the first 6 items explained 92% of the variance, 3 were the same as in the DOXA sample, while the other 3 were different but pertaining the same scale of the items present in the DOXA sample. The 6 original items ranked, indeed, in the first top-ten. In addition, the original 6 items explained more than the 90% of the variance of the longer index from the 22 item questionnaire. Finally, when the amount of variance explained was estimated in each sex and age strata, the figures ranged from 90.2 to 95.1%.

It is important to keep in mind that at the current development status the generalizability of the findings are exclusively confined to the Italian setting, and results cannot be transferred to other cultural and linguistic settings. We cannot exclude that additional analyses of foreign data from other countries could ultimately lead to a different item selection. Nonetheless, these results can be considered as a first step in the validation process of the PGWB-S, and as a promising starting point for future research on this matter.

## Declaration of the authors

The author(s) declare that they have no competing interests.

## Authors' contributions

EG & GA conceived the study, participated in its design and coordinate the manuscript drafting.

NG had a substantial role in writing the manuscript; PM helped in drafting the manuscript and participated in the design and coordination of development of the Italian version of PGWBI.

RC performed the statistical analysis and participated in preparing the manuscript; FP carried out one of the studies in patients; AC carried out the studies in students and helped in drafting the manuscript. All authors read and approved the final manuscript.

## Study funding

The study has been partially supported by Bracco SpA with a grant for the conduction of cross sectional population surveys by Doxa

## Supplementary Material

Additional File 1 Psychological General Well Being Index. Click here for file

## References

[B1] Hunt SM, McKenna S (1992). A British adaptation of the general Well-being Index: a new tool for clinical research. British J Med Economics.

[B2] Bullinger M, Heinisch M, Ludwig M, Geier S (1990). Skalen zur Erfassung des Wohlbefindens. Psychometrische Ueberpruefung des Profile of Mood States (POMS) und des Psychological General Well-Being Index (PGWBI). Z Differentielle Diagn Psychol.

[B3] Wiklund I, Karlberg J (1991). Evaluation of quality of life in clinical trials: selecting quality of life measures. Controlled Clin Trials.

[B4] Omvik P, Thaulow E, Herlan OB, Eide I, Midha R, Turner RR (1993). Double-blind, parallel, comparative study on quality of life during treatment with amlodipine or enalapril in mild or moderate hypertensive patients: a multicenter study. J Hypertens.

[B5] Walle PO, Westergren G, Dimenas E, Olofsson B, Albrektsen T (1994). Effects of 100 mg of controlled-release metoprolol and 100 mg of atenolol on blood pressure, central nervous system-related symptoms, and general well being. J Clin Pharmacol.

[B6] Rasmussen NA, Norholm V, Bech P (1999). The internal and external validity of the Psychological General Well-Being Schedule (PGWB). Quality of Life News Letter.

[B7] Wiklund I, Berg G, Hammar M, Karlberg J, Lindgren R, Sandin K (1992). Long-term effect of transdermal hormonal therapy on aspects of quality of life in postmenopausal women. Maturitas.

[B8] Havelund T, Lind T, Wiklund I, Glise H, Hernqvist H, Lauritsen K, Lundell L, Pedersen SA, Carlsson R, Junghard O, Stubberod A, Anker-Hansen O (1999). Quality of life in patients with heartburn but without esophagitis: effects of treatment with omeprazole. Am J Gastroenterol.

[B9] Reddy P, White CM, Dunn AB, Moyna NM, Thompson PD (2000). The effect of testosterone on health-related quality of life in elderly males – a pilot study. J Clin Pharm Ther.

[B10] Boman UW, Bryman I, Halling K, Moller A (2001). Women with Turner syndrome: psychological well-being, self-rated health and social life. J Psychosom Obstet Gynaecol.

[B11] Dupuy HJ, Wenger NK, Mattson ME, Furburg CD, Elinson J (1984). The Psychological General Well-being (PGWB) Index. Assessment of Quality of Life in Clinical Trials of Cardiovascular Therapies.

[B12] http://www.mapi-research-inst.com.

[B13] Grossi E, Mosconi P, Groth N, Niero M, Apolone G (2002). IL Questionario Psychological General Well Being. Questionario per la valutazione dello stato generale di benessere psicologico. Versione Italiana. Istituto di Ricerche Farmacologiche "Mario Negri", Milan.

[B14] Groth N, Cerutti R, Rivolta G, Grossi E (2001). Impact of transdermal estrogens treatment on postmenopausal symptoms and health-related quality of life: an Italian multicenter trial. J Hyg Prev Med.

[B15] http://crc.marionegri.it/qdv.

[B16] Apolone G, Mosconi P, Quattrociochi L, Gianicolo EAL, Groth N, Ware JE (2001). Questionario sullo stato di salute SF-12. Versione Italiana Guerini e Associati Editore, Milan.

[B17] Kodraliu G, Mosconi P, Groth N, Carmosino GC, Donzelli A, Gianicolo E, Rossi C, Apolone G (2001). Subjective health status assessment: evaluation of the Italian version of the SF-12 Health Survey. Results from the MiOS Project. J Epidemiol Biostatistics.

[B18] Ware JE, Kosinski M, Keller SD (1996). A 12-Item Short-Form Health Survey: Construction of scales and preliminary tests of reliability and validity. Med Care.

[B19] Gandek B, Ware JE, Aaronson NK, Apolone G, Bjorner JB, Brazier JE, Bullinger M, Kaasa S, Leplege A, Prieto L, Sullivan M (1988). Cross-validation of item selection and scoring for the Sf-12 Health Survey in nine countries: Results from the IQOLA Project. J Clin Epidemiol.

[B20] McHorney CA, Ware JE, Lu JFR, Sherbourne CD (1994). The MOS 36-Item Short-Form Health Survey (SF-36): III. Tests of data quality, scaling assumptions, and reliability across diverse patients groups. Med Care.

[B21] Ware JE, Gandek B (1988). Methods for testing data quality, scaling assumptions and reliability: The IQOLA Project approach. J Clin Epidemiol.

[B22] Stewart AL, Ware JE (1992). Measuring functioning and well-being: The Medical Outcomes Study Approach.

[B23] Aziz A, Bergquist C, Brannstrom M, Nordholm L, Silfverstolpe G (2005). Differences in aspects of personality and sexuality between perimenopausal women making different choices regarding prophylactic oophorectomy at elective hysterectomy. Acta Obstet Gynecol Scand.

[B24] Mannion AF, Elfering A, Staerkle R, Junge A, Grob D, Semmer NK, Jacobshagen N, Dvorak J, Boos N (2005). Outcome assessment in low back pain: how low can you go?. Eur Spine J.

[B25] Wiklund I, Karlberg J, Mattsson L (1993). Quality of life of postmenopausal women on a regimen of transdermal estradiol therapy: a double-blind placebo-controlled study. Am J Obstet Gynecol.

[B26] Wiklund I (1998). Methods of assessing the impact of climacteric complaints on quality of life. Maturitas.

[B27] Wool C, Cerutti R, Marquis P, Ciadella P, Herviè C, ISGQL (2000). Psychometric validation of two Italian quality of life questionnaires in menopausal women. Maturitas.

[B28] Dimenas E, Carlsson G, Glise H, Israelsson B, Wiklund I (1996). Relevance of norm values as part of the documentation of quality of life instruments for use in upper gastrointestinal disease. Scand J Gastroenterol Suppl.

[B29] Blomqvist A, Lonroth H, Dalenback J, Ruth M, Wiklund I, Lundell L (1996). Quality of life assessment after laparoscopic and open fundoplications. Results of a prospective, clinical study. Scand J Gastroenterol.

[B30] Revicki DA, Crawley JA, Zodet MW, Levine DS, Joelsson BO (1999). Complete resolution of heartburn symptoms and health-related quality of life in patients with gastro-oesophageal reflux disease. Aliment Pharmacol Ther.

[B31] Grossmann EM, Johnson FE, Virgo KS, Longo WE, Fossati R (2004). Follow-up of colorectal cancer patients after resection with curative intent-the GILDA trial. Surg Oncol.

