# Supplementary material for: Development and validation of the short version of the Psychological General Well-Being Index (PGWB-S)
Source: Health Qual Life Outcomes. 2006 Nov 14;4:88. doi: 10.1186/1477-7525-4-88 (PMC1647268; doi:10.1186/1477-7525-4-88)
Supplement: Additional File 1 — Psychological General Well Being Index. [file 1477-7525-4-88-S1.doc]

# **Additional file Psycological General Well Being Index**

**1. How have you been feeling in general during the past month?**

In excellent spirits………………………………………………………………… _5_

In very good spirits……………………………………………………………….. _4_

In good spirits mostly…………………………………………………………….. _3_

I have been up and down in spirits a lot………………………………………….. _2_

In low spirits mostly……………………………………………………………… _1_

In very low spirits………………………………………………………………… _0_

**2. How often were you bothered by any illness, bodily disorder, aches or pains**

**during the past month?**

Every day………………………………………………………………………… _0_

Almost every day………………………………………………………………… _1_

About half of the time……………………………………………………………. _2_

Now and then, but less than half the time………………………………………... _3_

Rarely…………………………………………………………………………….. _4_

None of the time………………………………………………………………….. _5_

**3. Did you feel depressed during the past month?**

Yes – to the point that I felt like taking my life………………………………….. _0_

Yes – to the point that I did not care about anything…………………………….. _1_

Yes – very depressed almost every day………………………………………….. _2_

Yes – quite depressed several times……………………………………….……... _3_

Yes – a little depressed now and then……………………………………………. _4_

No – never felt depressed at all…………………………………………………… _5_

**4. Have you been in firm control of your behaviour, thoughts, emotions**

**or feelings during the past month?**

Yes, definitely so………………………………………………………………… _5_

Yes, for the most part……………………………………………………………. _4_

Generally so……………………………………………………………………… _3_

Not too well……………………………………….……………………………... _2_

No, and I am somewhat disturbed……………………………………………….. _1_

No, and I am very disturbed……………………………………………………… _0_

**5. Have you been bothered by nervousness or your “nerves” during**

**the past month?**

Extremely so – to the point where I could not work or take care of things……… _0_

Very much so…………………………………………………………………….. _1_

Quite a bit………………………………………………………………………… _2_

Some – enough to bother me…………………….………………………………. _3_

A little……………………………………………………………………………. _4_

Not at all………………………………………………………………………….. _5_

**6. How much energy, pep, or vitality did you have or feel during**

**the past month?**

Very full of energy – lots of pep…………………………………………………. _5_

Fairly energetic most of the time……………………………………………….… _4_

My energy level varied quite a bit………………………………………………... _3_

Generally low in energy or pep………………….…………………………….…. _2_

Very low in energy or pep most of the time……………………………………… _1_

No energy or pep at all – I fell drained, sapped…………………………………... _0_

**7. I felt downhearted and blue during the past month.**

None of this time………………………………………………………………… _5_

A little of the time……………………………………………….……………….. _4_

Some of the time……………………………………………….………………… _3_

A good bit of the time………………….…………………………….…………... _2_

Most of the time………………………………………………………………….. _1_

All of the time………………………………….………………………………… _0_

**8. Were you generally tense or did you feel any tension during the past month?**

Yes – extremely tens, most or all of the time……………………………………. _0_

Yes – very tense most of the time……………………………………………….. _1_

Not generally tense, but did feel fairly tense several times……………………….. _2_

I felt a little tense a few times………………….………………………………… _3_

My general tension level was quite low…………………………………………. _4_

I never felt tense or any tension at all…………………………………………….. _5_

**9. How happy, satisfied, or pleased have you been with your personal life**

**during the past month?**

Extremely happy – could not have been more satisfied or pleased……………… _5_

Very happy most of the time…………………………………………………….. _4_

Generally satisfied, pleased……………………………………………………… _3_

Sometimes fairly happy, sometimes fairly unhappy…………………………….. _2_

Generally dissatisfied or unhappy…….…………………………………………. _1_

Very dissatisfied or unhappy most or all the time……………………………….. _0_

**10. Did you feel healthy enough to carry out the things you like to do**

**or had to do during the past month?**

Yes – definitely so………………………………………………..……………… _5_

For the most part…………..……………………………………….…………….. _4_

Health problems limited me in some important ways…………………………… _3_

I was only healthy enough to take care of myself …………………………….. _2_

I needed some help in taking care of myself.……………………………………. _1_

I needed someone to help me with most or all of the things I had to do..……….. _0_

**11. Have you felt so sad, discouraged, hopeless, or had so many problems**

**that you wondered if anything was worthwhile during the past month?**

Extremely so – to the point that I have just about given up..……..……………… _0_

Very much so….…………..……………………………………….…………….. _1_

Quite a bit………………………………………………………………………… _2_

Some – enough to bother me…………………………………………………….. _3_

A little bit…………………………………..……….……………………………. _4_

Not at all………………………………………………………………....……….. _5_

**12.** **I woke up feeling fresh and rested during the past month.**

None of the time…………………………………………....……..……………… _0_

A little of the time..………..……………………………………….…………….. _1_

Some of the time..………………………………………………………………… _2_

A good bit of the time…………………………………………………………….. _3_

Most of the time…...………………………..……….……………………………. _4_

All of the time…………………………………………………………....……….. _5_

**13. Have you been concerned, worried, or had any fears about your health**

**during the past month?**

Extremely so…..…………………………………………....……..……………… _0_

Very much so……..………..……………………………………….……………. _1_

Quite a bit……....………………………………………………………………… _2_

Some, but not a lot….……………………………………………………………. _3_

Practically never......………………………..……….……………………………. _4_

Not at all………………………………………………………………....……….. _5_

**14. Have you had any reason to wonder if you were losing your mind,**

**or losing control over the way you act, talk, think, feel or of your**

**memory during the past month?**

Not at all…..…………………………………………....……..…………………. _5_

Only a little……..………..……………………………………….…………….... _4_

Some – but not enough to be concerned or worried about………………………. _3_

Some and I have been a little concerned………………………………………… _2_

Some and I am quite concerned..…………..……….……………………………. _1_

Yes, very much so and I am very concerned…………………………....……….. _0_

**15. My daily life was full of things that were interesting to me during**

**the past month.**

None of the time……………………………………....……..…………………... _0_

A little of the time………..……………………………………….…………….... _1_

Some of the time…………………………………………………………………. _2_

A good bit of the time………………….………………………………………… _3_

Most of the time………………..…………..……….……………………………. _4_

All of the time………………………………………………………....…………. _5_

**16. Did you feel active, vigorous, or dull, sluggish during the past month?**

Very active, vigorous every day……..………………....……..…………………. _5_

Mostly active, vigorous – never really dull, sluggish…………….…………….... _4_

Fairly active, vigorous – seldom dull, sluggish………….………………………. _3_

Fairly dull, sluggish – seldom active, vigorous..………………………………… _2_

Most dull, sluggish – never really active, vigorous...……………………………. _1_

Very dull, sluggish every day…………………………………………....………. _0_

**17. Have you been anxious, worried, or upset during the past month?**

Extremely so – to the point of being sick or almost sick……..………………….. _0_

Very much so…...………..……………………………………….…………….... _1_

Quite a bit……...…………………………………………………………………. _2_

Some – enough to bother me..………….………………………………………… _3_

A little bit……..………………..…………..……….……………………………. _4_

Not at all…….………………………………………………………....…………. _5_

**18. I was emotionally stable and sure of myself during the past month.**

None of the time……………………………………....……..…………………... _0_

A little of the time………..……………………………………….…………….... _1_

Some of the time…………………………………………………………………. _2_

A good bit of the time………………….………………………………………… _3_

Most of the time………………..…………..……….……………………………. _4_

All of the time………………………………………………………....…………. _5_

**19. Did you feel relaxed, at ease or high strung, tight, or keyed-up**

**during the past month?**

Felt relaxed and at ease the whole month……………………..………………….. _5_

Felt relaxed and at ease most of the time………………………….…………….... _4_

Generally felt relaxed but at times felt fairly high strung...………………………. _3_

Generally felt high strung but at times felt fairly relaxed………………………… _2_

Felt high strung, tight, or keyed-up most of the time..……………………………. _1_

Felt high strung, tight, or keyed-up the whole month.………………....…………. _0_

**20. I felt cheerful, lighthearted during the past month.**

None of the time……………………………………....……..…………………... _0_

A little of the time………..……………………………………….…………….... _1_

Some of the time…………………………………………………………………. _2_

A good bit of the time………………….………………………………………… _3_

Most of the time………………..…………..……….……………………………. _4_

All of the time………………………………………………………....…………. _5_

**21. I felt tired, worn out, used up, or exhausted during the past month.**

None of the time……………………………………....……..…………………... _5_

A little of the time………..……………………………………….…………….... _4_

Some of the time…………………………………………………………………. _3_

A good bit of the time………………….………………………………………… _2_

Most of the time………………..…………..……….……………………………. _1_

All of the time………………………………………………………....…………. _0_

**22. Have you been under or felt you were under any strain, stress, or pressure**

**during the past month?**

Yes – almost more than I could bear or stand………………..………………….. _0_

Yes – quite a bit of pressure…………..………………………….…………….... _1_

Yes, some – more than usual………………………….....………………………. _2_

Yes, some – but about usual……………………………………………………... _3_

Yes – a little………………………………………...……………………………. _4_

Not at all…………………………………………….………………....…………. _5_
